# Essays on the Philosophy of Vitality, as Contradistinguished from Chemical and Mechanical Philosophy, and on the Modus Operandi of Remedial Agents

**Published:** 1843-01

**Authors:** 


### Art. XII.

1. *Essays on the Philosophy of Vitality, as contradistinguished from Chemical and Mechanical Philosophy, and on the modus operandi of Remedial Agents.* By Martyn Paine, A.M. M.D., Professor of the Institutes of Medicine and Materia Medica in the University of New York, &c. &c.—*New York*, 1842. 8vo, pp. 70.

2. *The Cyclopædia of Anatomy and Physiology.* Edited by Robert B. Todd, M.D. F.R.S., Professor of General Anatomy and Physiology in King's College, London—Article "*Life*," by William B. Carpenter, M.D., Lecturer on Physiology in the Bristol Medical School.—*London*, 1840. 8vo, pp. 20.

The usual personalities directed against us, which the first of these pamphlets contains, would not alone prompt our notice of it; since we have no inclination to carry on a contest with an adversary who cannot give us the credit either of gentlemanly feeling, or honesty of purpose. We shall take the opportunity, however, of briefly noting the present state of the questions at issue between us; for the information of such of our readers as care to learn it, without wading through the numerous pages in which it is mystified—and misty-fied—by Dr. Paine. The learned gentleman and ourselves were on the best possible terms, until the unlucky hour when we unfavorably reviewed his Commentaries. This is evident, from the very complimentary testimonial he had given in favour of our Journal, not long before that review appeared;* and from the frequent and gratifying allusions he makes in his Commentaries to various preceding articles in it. We could have had no other motive, then, but a sense of justice, in passing upon it the verdict we did. Pledged as we are to the public, to do justice without fear or favour, we used towards Dr. Paine the same impartiality which we have exercised with reference to individuals who rank far above him as medical philosophers; and that the opinion we expressed was not an exception to the general verdict of the well-informed part of the profession, is apparent from the fact, that reviews equally unfavorable, some even more unfavorable, appeared in various other most respectable journals of this country, Germany, and America. From his first glance at our review, the whole current of Dr. Paine's thoughts towards us seemed to be changed. Instantly, as if "perplexed in the extreme" like Othello, in his jaundiced apprehension

"*Our* name, that was as fresh
As Dian's visage, is now begrimed and black."

We are no longer the able and impartial journalists we once were; but scarcely any language is strong enough to express the grossness of our misrepresentations, the darkness of our ignorance, the depth of our malignity. And yet we think our readers will not have perceived any such change in the tone of our journal as may confirm Dr. Paine's imputations; and may possibly incline to think that the change is solely in his own mind, and that his wounded vanity has a large share in producing it. To Dr. Paine's exposition of our errors of criticism, we have made no reply, and do not intend to make any; simply because if any of his statements are to be answered, the whole must be; and as Dr. Paine's *Examen* of our review was about four times the bulk of the original, OUR *Examen* of HIS *Examen* would be swollen from an overgrown pamphlet into a goodly volume, the production of which would involve an amount of trouble which *we* do not consider at all commensurate with the importance of the subject—being satisfied that, as Dr. Paine's bulky pamphlet has been but little read, our

* As the record of a rare psychological phenomenon, when taken in conjunction with the subsequent denouncements of the writer, we here give permanency to the document referred to; it may hereafter be useful to the metaphysical inquirer.

"*New York; August* 19, 1840.

"The subscriber, having read with attention the British and Foreign Medical Review as far as published, would commend this journal to such of his professional brethren as may not be familiar with its merits, as abounding with the latest information upon medical topics and collateral branches, gleaned from all parts where knowledge is cultivated. The critical articles are of the highest order; emanating from erudite genius, liberal and generous, yet devoted to the paramount interests of science. Its range of observation is so extensive, and its critical articles so elaborate, it may be said, without interfering with the interests of other medical periodicals at home and abroad, that this journal is indispensable to all who would most improve their acquaintance with philosophical medicine, or practice the art in its most rational aspects.

"MARTYN PAINE, M.D."

volume would excite still less attention, and that our readers will form their judgment of the validity of Dr. Paine's charges, rather from our general character for impartiality and competent knowledge of the subjects we treat of, than from an investigation of their details. To *either* source of judgment, however, we fearlessly appeal.

Dr. Paine's "Examination," however, contained a specific charge, totally distinct from those relating to himself, (though he has done his best to mix the two together,) seriously implicating the character of a gentleman known to be connected with this Journal. This being placed in a form adapted to strike the eye of every one who opened the pamphlet, gained very extensive currency; and, from certain internal probabilities, as well as from the very artful mode in which the evidence was brought forwards by Dr. Paine, the charge was readily credited by those who were not acquainted with the personal character of the individual attacked. He gave it at once, however, a positive denial; and appealed to the Editor of this Journal for his confirmation. That confirmation was given promptly and without the slightest reservation, because there was nothing to reserve. The charge was admitted to be true in regard to the writer of a certain *Review* (that of Hunter's works) in this Journal; but that writer was *not* the individual to whom it was attributed by Dr. Paine. And although it may suit Dr. Paine's purpose to treat these counter-statements with a sneer, and to trust more to his own dogmatic *assumptions*, than to the positive assertions of two individuals of respectable name, regarding a matter of which they alone can *know* the real truth, we feel sufficiently confident of the good feeling and candid judgment of our professional brethren to leave the matter in their hands, without taking the trouble to produce that further evidence of the falsity of Dr. Paine's charge, which it would be easy for us to obtain. We shall only add that the assertion which Dr. Paine gives in his preface to the present treatise, on the information of a "sure source in Europe, that Dr. Forbes has stated that Dr. Carpenter wrote a part of the review of my Commentaries, and the plagiarist the other part," is as false as Dr. Paine's other assertions on the same subject; since Dr. Forbes *never* has made any such statement, for the very simple reason that he *never could* have made it, the fact being quite otherwise.*

With these remarks we shall close the controversy, so far as *we* are concerned. Dr. Paine may continue his attacks upon us, as long as he finds it conducive to his reputation, or beneficial to his pocket, to do so; but we believe that we shall consult our own best interests by leaving them unanswered.

We shall now proceed to examine the scientific portion of Dr. Paine's volume, which we are the more willing to do, inasmuch as we do not recollect any previous occasion on which we have given a formal exposition of

* We will just acquaint Dr. Paine with a small fact, ("these little things are great to little men,") which may gratify him as helping to explain some of his odd mistakes and strange flounderings about the truth, in his early as well as in his later reclamations, and the withholding of which at the time was, we fear, partly owing to our wicked indulgence of the Lucretian satisfaction (*Suave mare magnum*, &c.) of witnessing in safety from our editorial watch-tower his dangerous and unwieldy gambols on the sea of criticism; it is this: the *Article* in which the plagiarism appeared was composed of *three* papers, written by *two* different persons, united into *one article* by the editor. Dr. Paine seems marvellously ignorant of the universally known *secrets* of journalism.—Ed.

our views on the "Philosophy of Vitality." Agreeing as we do on this subject with the author of the second of the treatises of which the titles head this article, we consider that our object may be best accomplished by giving a brief analysis of his views; and by then inquiring how far Dr. Paine's representations of them, or strictures upon them, are well founded.

Every physiological writer is entitled to give his definition of LIFE; since it is impossible to conduct any reasoning upon a subject of such abstract character, unless the precise meaning of the terms employed be fixed at starting. The definition here given by Dr. Carpenter has special reference to the definition of the term DEATH, given by Dr. Symonds in a former number of the same Cyclopædia, and is designed to be antagonistic of it. As death is the condition of an organized body in which its distinguishing properties have been abolished, and its peculiar actions have ceased, so life is "the state of action peculiar to an organized body or organism." Between these two conditions, is that state in which there is no obvious functional activity; yet the peculiar properties of the organism are not absent, and only need the requisite stimuli to call them into operation: this is the condition of the seed and of the torpid animal; and it is designated by Dr. Carpenter as that of *dormant vitality*. We will not say that this definition of life is incapable of improvement; but we do not see how it can be made more precise, without involving a great extension of its terms. It depends upon the known acceptation of the words "organized body or organism;" and as these are not subject (so far as we are aware,) to any doubt, we need not commence our exposition by a discussion of their meaning.

No one in his senses can fail to perceive the striking differences between the actions of organized beings, and those of brute matter. The phenomena of sensation, thought, and spontaneous movement, which are exhibited by animals alone, are obviously of a character totally distinct from any which we witness in the inorganic world; and the acts of nutrition and reproduction, which are common to all classes of living beings, have but a very remote analogy, if any, to the physical or chemical operations of inanimate bodies. But these phenomena are to be studied *in the same manner* as those of physics or chemistry; that is, we must apply to them the principles of the inductive philosophy; and, by the collection and comparison of instances, must strive to refer them to general principles or laws. But when we speak of such general principles or laws, which we regard as the ultimate facts of science (as for instance the principle or law of gravitation), we must carefully bear in mind that nothing more is meant by them, than concise expressions of the mode in which the Creator operates on matter,—expressions which at once imply the uniformity of the operation and the universality of its extent. Thus the law of gravitation, the most general yet discovered, is merely an expression of the fact that, under all circumstances whatever, masses of matter have an attraction for each other in direct proportion to their bulks, and in the inverse ratio of the square of their distance. This law, then, is founded on a property which pervades all matter, and is essentially connected in our minds with the idea of materiality. The property can only manifest itself, however, when certain conditions of action are supplied; thus, if only one mass of matter existed in the universe, we should not be cognizant that it possessed the power of attracting another. A similar illustration may be drawn from physiology. There is a certain peculiar form of tissue, to which we give the name of muscular fibre; and this tissue possesses the property of contracting upon the application of particular stimuli, provided certain conditions are supplied. This property is so essential to our idea of the substance known as muscular fibre, that even if we were to find a tissue (which we do in no instance,) apparently presenting all its external characters, but destitute of this property, we should at once conclude that it must differ in some essential particular of its structure and composition from real muscle. Further, we do not find this property existing in any tissue which does not possess the particular structure which we denominate muscular; and we never find it lost by the structure which once possessed it, until the cessation of the supply of arterial blood deprives it of one of the conditions necessary for the manifestation of its property; or unless an alteration in its nutrition, or incipient decay, have produced a change in its structure or composition. Hence the idea of contraction on the application of a stimulus, is associated in our minds with that organized fabric which we term muscular fibre; just as explosive power, on the application of flame, is associated with that mixture of sulphur, charcoal, and nitre, which we call gunpowder. An amount of decomposition inappreciable by our present means of observation may destroy the contractility of muscle; just as a very small amount of dampness, the existence of which we might only know by its effects, impairs the combustibility of gunpowder. But there is this fundamental difference between the two cases, which at once proves that there is no further relation between them than one of analogy. The explosive force of gunpowder is produced by the mode in which its different components act upon one another, when flame is applied, according to principles strictly *chemical*;—the chemist makes the mixture, and adjusts the proportions in such a manner as to produce the most advantageous result, by the known tendency of the ingredients to form certain new combinations according to definite proportions. But no chemical admixture of ingredients, nor any structural arrangement of them that the physical philosopher might devise, could produce a tissue that would contract on the application of a stimulus. In its origin muscular fibre is entirely distinct from any inorganic compound, for it can only be generated by a living organism; and it is thus equally alien, in its structure and its properties, from any possible aggregation of inorganic matter. Hence to its structure we apply the term *organized*, indicative of that peculiar arrangement, which we never see in brute matter, and which we know, from experience, can only be given by the action of a pre-existing organism; and to its properties we give the term *vital*, indicative of a class, or category, entirely distinct from physical or chemical, as these are from each other. In the same manner we might proceed in regard to all the other tissues of the living body; we should find that each has its peculiar structure and peculiar properties; and that certain vital properties are inseparably connected with certain forms of organized tissue. True it is that we find different modifications of the same kind of property possessed by tissues *apparently* the same; as, for example, the secreting cells of one gland do not present any obvious differences from those of another, though their products are so unlike; or we find some cells undergoing transformation into nervous tissue, whilst others, not at first distinguishable from them, are changed into muscular fibre. But will any microscopist or chemist be bold enough to say, that he is so cognizant of *all* the characters presented by these several tissues, as to feel certain of their identity of structure and composition? We should hope not.

We have heard of no physiologist of modern times possessing any claim to scientific distinction, who does not fully recognize the existence of a set of *vital* properties, totally distinct from those of a physical or chemical nature, and exhibited only by *organized* structures. In our estimation, no absurdity could be greater than to deny them; and we challenge Dr. Paine, or any other captious *hyper-vitalist*, to prove that we have ever done so; not by select sentences isolated from their context and pieced together so as to present a meaning quite contrary to that which they were intended to convey; but by legitimate quotations.

But there are many shades of opinion amongst physiologists, as to the first origin of these vital properties; and as to the greater or less exclusiveness of their operation in the living body. Each of these questions we shall briefly notice.

First, then, as to the origin of vital properties. We see these so inseparably connected with particular forms of structure,—the properties never being manifested without the structure, and the structure never existing in its perfect state, and in its normal conditions, without the properties,—that no reasonable ground can be assigned for attributing vital properties to anything else than that peculiar structural arrangement, and that peculiar union of elements, which characterize the tissue exhibiting them. There is here a strong *analogy*, then, to the production of a new and peculiar set of properties in a piece of mechanism or in a chemical compound, by a certain disposition of elements; but it is *only* an analogy, since the conditions requisite for the production of an organized tissue are, as already shown, of a new and peculiar character. Still the analogy is important in this,—that, as we do not consider it necessary to imagine the separate existence of an *elastic principle* which is imparted to steel when tempered in a peculiar way,—or of a *saline principle* which is imparted to muriatic acid and soda in the act of their combination,—so there is no necessity to infer the existence of a *vital principle*, because a tissue formed in a peculiar manner possesses peculiar properties. And upon the logical principle of avoiding unnecessary hypotheses, we cannot but do wrong in making such an assumption. For we may consider it as a law of the Creator, equally constant in its operation with any of those already alluded to, that the act of organization, or the production of an organized structure out of an amorphous *plasma*, does generate or develope certain properties, which are as closely related to the structure as elasticity is to blue-tempered steel, or saline taste to muriate of soda. If there be not such laws of uniform operation in physiology, we know not what hope there is of ever raising it in the scale of sciences.

Now if the validity of this reasoning be admitted, there is only one step further back to be made, towards what we regard as the true theory of the vital properties. If the peculiar properties possessed by a certain tissue are to be attributed to that peculiarity of its structure and composition, which is termed its organization, we must attribute the capability of undergoing this organization to the elements of which it is composed; in other words, we must regard them as possessing *vital* properties in a dormant or inactive condition, just as they possess *physical* and *chemical* properties, which do not manifest themselves until the requisite conditions are supplied. Thus we know by experience, that oxygen is a supporter of combustion; but who would have learned this from its sensible properties, or from the mode in which it is generated? We know from experience that iron is capable of being rendered magnetic; and yet for how many ages had mankind been using iron before this capability was discovered, simply because the requisite conditions for its manifestation were not supplied? Just in the same manner, we know that oxygen, hydrogen, carbon, and nitrogen, united in a peculiar mode, produce a compound having peculiar properties, of which we should not have guessed the existence if we had not learned them by experience; the mode of union is peculiar, and not imitable by the physical or chemical philosopher; the properties are also peculiar, but they are not less the result of that act of combination and arrangement. We think it a strictly logical deduction, then, that all those forms of matter which enter into the composition of an organized body possess vital properties, which, like any other properties, manifest themselves as soon as the requisite conditions are supplied. To this view Liebig has recently been led, as we pointed out in our last volume, (p. 496,) by a train of reasoning which was doubtless original with himself, although by no means new to us; and, as he justly remarks, "it takes from the vital phenomena nothing of their wonderful peculiarity, whilst it may be considered as a starting point from which an investigation into these phenomena and the laws which regulate them may be commenced."

If the train of reasoning we have just sketched be correct, and some very able logicians have expressed their inability to detect any flaw in it, the development of a living organism from a germ is the necessary result of the vital properties of that germ, which enable it to assimilate and organize the nutriment supplied to it, and thus to call into operation the properties which were previously dormant in its elements; and the whole maintenance of that organism is an action of the same kind, involving as it does a continual appropriation of fresh nutriment to repair its waste, a continual production of fresh tissue in place of that which has undergone decay, and therefore a continual development of vital properties, whose actions shall replace those which have ceased from the loss of the necessary conditions. But whence the vital properties of the germ? They are derived from the parent that produced it. There is something most wonderful, we admit, in the fact, that the mere nucleus of a cell, placed in a certain position favorable to its evolution, should become a moving, feeling, thinking being: but the phenomenon is only more wonderful in degree, not in kind, than the growth of the first simple cell, which may remain as the humble fungus, from a similar germ. Both are instances of the operation of properties purely vital, according to laws which must have been impressed on the material elements concerned, at the time when they were first called into existence by the *fiat* of their Creator. Dr. Paine makes an extraordinary use of the doctrines entertained by Dr. Prichard and Dr. Carpenter on this subject, which leads us to suppose that his opinions on the divine agency are very peculiar. Thus he states that "both of these writers see so much of peculiar design in organic nature, that they find it impossible to interpret the phenomena of organic beings upon the chemical and physical principles which they have so strenuously put forth, and in the end assign them to the *immediate* action of the Deity." After two quotations from Dr. Prichard, he continues: "This is a far greater admission than the vitalist can desire, since, if the development and growth of the germ depend immediately upon Almighty Power, so must all the analogous processes of the living being at all stages of its existence. But whilst this doctrine is utterly exclusive of all the assumed chemical agencies at all periods of life, and overlooks the analogy between the development of the germ and the subsequent processes, there can be no hesitation as to the disposition which should be made of it, without any reference to its prevaricating nature." So then, according to Dr. Paine, to acknowledge the immediate agency of the Deity in any class of phenomena, is to remove them from the pale of chemical and physical laws; or to say that vital phenomena are the results of the immediate action of the Deity, is to affirm that they are not under the dominion of laws analogous to (but not identical with) those of physics and chemistry: as if these laws were in *any* case aught else than expressions of the mode in which the Creator is constantly and immediately operating on every particle of matter in the universe. Dr. Prichard's object is to show that there is no *intermediate agent*, interposed between the Deity and the living organism, any more than there is an agent governing the solar system or any other example equally indicative of design in its adjustment and of foreknown harmony in its operation; and the same line of argument has been carried out, as we think successfully, by Dr. Carpenter. (See Brit. and For. Med. Rev., vol. VII. p. 172.)

We briefly sum up this portion of the subject, therefore, by affirming our belief that, as there are certain properties of matter, which operate in a certain uniform manner, (or according to certain laws,) to produce the class of phenomena which we ordinarily designate as physical,—and as there are certain other properties which act in *their* peculiar manner to produce chemical phenomena—so there is another class, restricted to particular elements, which, when operating under certain required conditions, produce vital phenomena. All these phenomena, resulting from the action of properties essential to the constitution of the several forms of matter, according to laws pre-determined by the Deity, are equally the results of His agency; and nothing is to be gained by imagining that there is any delegation to inferior or secondary powers. Such delegation must be understood, if the term *vital principle* be employed in any other sense than as a comprehensive expression of the vital properties of the organism.

II. The question that remains for consideration relates to the extent to which the operations of the living organism are the result of its purely *vital* properties; or how far the *chemical* and *physical* properties of its materials are concerned in its actions. To deny that the latter participate in them would seem to us the height of absurdity. Is the force generated by the vital contractility of the muscle, applied in moving the bone, on any other than strictly mechanical principles? Or does the movement of the blood through the large vessels take place in a mode different from that in which a similar fluid would move in an apparatus of inorganic tubes similarly constructed, and under the influence of forces similarly applied, although generated by a piece of human mechanism? Or do an acid and alkali brought together in the stomach neutralize each other less *there* than anywhere else? We cannot conceive of any hesitation in the reply to such questions. How far, then, do physics and chemistry take a share in the maintenance of the life of the organism? With regard to chemistry, we briefly expressed an opinion, in our review of Liebig's work, which appears to us to embody the whole principle of the matter; and as it is brief, we shall introduce it here with the addition of one or two illustrations. "Chemistry may fairly take cognizance of all those processes by which the vegetative functions of the living organism are brought into relation with the world around. It may on the one hand trace the conversion of the alimentary materials into organizable products; but there it ends: it can throw no light on the process of organization, or upon the new (vital) properties which are called forth by it. On the other hand, it can take up the same materials, in the act of being restored to the inorganic world by the metamorphosis of the tissues; and it may give an account of all the changes which the materials undergo up to the time when they are finally cast off by some of the excretory processes. But of the *vital* functions of organized tissues, which it is the peculiar province of the physiologist to investigate, chemistry can give no account whatever." Thus, the plant takes up from the soil and from the atmosphere three binary compounds—water, carbonic acid, and ammonia, besides certain mineral ingredients which we need not at present consider. Out of these it elaborates certain ternary compounds destitute of azote—as gum, sugar, and oil; and certain quarternary compounds containing azote, and identical in composition with the albumen and fibrin of animals. Now it is a well-ascertained fact, that the *tissues* of plants are composed of the non-azotized compounds only, which are formed by all vegetables; whilst the azotized products are merely deposited in the cells and lacunæ of the fabric. Gum may be regarded as the organizable principle of plants; for the sugar and oily matter which they largely contain, seem to pass through the condition of gum before they can be converted into organized tissue. These principles are well known to have strong chemical relations; so that we can convert either gum, starch (which is gum undergoing organization), or even organized tissue, into sugar, by chemical agencies only. Now we do not entertain much doubt, that they will in time be found capable of being formed by the union of their elements; indeed with regard to fatty matter this has already been accomplished. But the conversion of any of them into an organized tissue, possessed of vital properties, is a change which can only be effected by a previously-existing organism in a state of vital activity. Again, the azotized products found largely in certain plants, are not, so far as they are concerned, organizable principles, but are rather to be considered as secretions; for there is no evidence that they ever enter into the composition of vegetable tissue. In regard to these also, we think no one is warranted in affirming that they are aught else than chemical compounds; or that, if we knew the precise mode in which their elements are brought together, and could imitate it, we should be unable to obtain the same product. Now these azotized compounds secreted by plants are the organizable principles of animals, being used by them as the materials for the construction of their tissues. There is no evidence that the non-azotized compounds of plants which are taken into the animal system ever undergo organization; they are destined either to sustain the respiration, or to be cast off by other secretions, or to be deposited as fatty matter.

The process of digestion is nothing else, in our view, than the chemical reduction of these two classes of principles to a form in which they can be most readily absorbed and applied to the purposes of the economy; and we believe that there are few, if any, intelligent physiologists (we are sorry that we cannot include Dr. Paine in this category) who do not entertain the same opinion. Dr. Paine asked rather triumphantly, in his "Commentaries," whether albumen had ever been produced by artificial digestion. We are now in a position to give him a more positive reply than we could at that time offer;—namely, that albumen is not *produced* in natural digestion, being merely *reduced*, from the solid condition in which it existed in the articles of food taken into the stomach, to the fluid form; and that this reduction may be effected by artificial, as completely as by natural digestion, although with less rapidity, because it is impossible exactly to imitate all the physical and chemical operations to which the food is subjected in the stomach. We believe, therefore, that the operations of vitality commence only with the absorption of the alimentary materials thus prepared. The albumen is gradually converted, under the influence of the living tissues over which it passes, into the semi-vitalized fibrin; and this is converted, under the same influence, into the various solid tissues possessed of their respective vital properties. This conversion is an act of vitality alone, which physics and chemistry have nothing to do with, otherwise than as furnishing the materials for it. When these tissues have performed their appointed functions, and lived out their assigned period, they undergo decay; and their products are partly destined to be thrown off by secretion, and partly (we are inclined to believe) again to contribute to the nutrition of the system; the portions fit for the latter purpose being taken up and conveyed back into the circulation by the lymphatic system. Now that the products of excretion are capable of being formed by chemical agencies alone, may be inferred from the well-known fact that urea has been artificially generated by the union of its components. The objection which might for some time have been brought against such a statement,—namely, that *cyanogen*, which is essentially concerned in this manufacture, is, though a binary compound, only procurable by the decomposition of animal tissue, and that any compound into which it may enter is still, in some sense, of an organic nature,—has been recently removed by the artificial production of this substance from its elements, carbon and nitrogen.

We trust that we have now stated our views on this subject with sufficient precision to render it unnecessary to dilate further upon them. We have shown that as the physical and chemical sciences are concerned with two classes of properties of matter (which, so far as we at present know, are distinct from each other,) the science of physiology is essentially different from either of them, in that it is based on the operation of a set of properties, not possessed by all elementary substances, and only manifested under certain peculiar conditions, belonging therefore to a category entirely distinct, to which we give the appellation of vital. But as in the operation of any complex piece of mechanism of human construction, such as the steam-engine, we find a variety of principles, chemical and physical, brought into action with one common end,—so in the living organism (constituting as it does but a part of the great system of the universe, subjected to its laws, and unable to exist but by its various relations with the inorganic world around,) there is a like combination of physical, chemical, and vital operations, having all one common purpose, and mutually adjusted by the all-wise Designer, in such a manner as to harmonise with each other in maintaining that state of activity of the entire system which is called its Life.

We shall now offer a few remarks on Dr. Paine's Treatise, some quotations from which will, we think, satisfy our readers as to its object and merits. The following is from the preface.

"The tendency of the labours now in progress in organic chemistry, to the subversion of physiological science, and therefore of pathological and therapeutical principles, and, as another necessary consequence, of rational practice, induces me to persevere in contributing my humble efforts to counteract the influence of the iatro-chemical philosophers. However laudable may be their motives, and however they may astonish us with revelations in inorganic chemistry, and multiply the sources of human happiness, they are nevertheless employed, in their interpretation of 'facts' relative to organic chemistry, in opposition to the experimental results which have been for ever in undeviating progress in every individual of the organic kingdoms. The enchantments, however, of a fascinating pursuit, and an imperfect acquaintance with those profound institutions of nature which are entirely foreign to the laboratory, and which can only be known through the accumulated inquiries of ages into both the natural and morbid phenomena of organic beings, must be allowed the weight of an apology. But an attempt to overthrow the experience of the past, and to obscure what is written in the most legible characters, and, as it were, in thousands of languages, upon the tablets of organic life, by a distorted construction of '*facts*,' which are yielded by test-glasses and crucibles, appears to me to be an enterprise which should alarm physiologists as to its pernicious consequences. That we are on the eve of some vast convulsion in physiology can no more be doubted, by those who watch the signs of the times, than that the declaration has been promulgated, *ex cathedrâ*, that 'medicine is now in its infancy,' or that efforts were made to establish a spurious system upon that assumption." (p. vi.)

Dr. Paine seems to forget that "truth never can be opposed to truth;" and that the inductions of organic chemists, respecting some of the phenomena which occur in the living organism, *may possibly* be more correct than *his* interpretation of the "experimental results which have been for ever in undeviating progress in every individual of the organic kingdoms." A man who sets out with the confidence that he must be right, and that every one who differs from him must be wrong, is not the sort of guide we should select in the investigation of the relative bearings of organic chemistry and physiology.

The professed object of Dr. Paine's first essay,—that on vitality as contradistinguished from chemical and mechanical philosophy,—is to state "some original views relative to the doctrines of life, as contradistinguished from those which respect inorganic nature. These doctrines lie at the foundation of all medical philosophy, of all practical medicine." This he does on the following plan:

"I shall proceed, in the first place, to carry out the illustration of an important and fundamental principle of nature, as briefly set forth in my 'Examination of Reviews.' This exposition consists in a deduction of the doctrines of the vitalists from the phenomena that attend the development of the incubated egg. It is also one of my present objects to extend the application of the foregoing principle, and to consider—

"1st. The constitutional nature of the ovum.

"2d. To show farther by the philosophy of generation, and by the nature of the powers which are universally admitted to be alone concerned in developing the germ or ovum, and in forming the organs of the new being, that the *same* powers are, also, *alone* concerned in carrying on for ever afterwards the processes of life, and, of course, that no new powers, or principles, are introduced.

"3d. To consider the manner in which the germ is impregnated, or its vital properties so stimulated into action, as to result in the development of the germ and in unfolding the various attributes of the new being.

"4th. To show that we may find in the physiology of generation, or the principles through which the ovum is impregnated, the whole philosophy of organic life, or the principles through which the actions of life are for ever carried on.

"5th. To state the manner in which the natural peculiarities of each parent, whether as it respects the properties of life, or the physical conformation, are infused into the germ, and combined in the full-grown offspring.

"6th. To show that hereditary diseases are transmitted in the same way as those more natural peculiarities which belong to parents.

"7th. To show, also, that the principles which are concerned in the transmission of hereditary diseases are the same as concur in the production of ordinary diseases.

"8th. To deduce from the philosophy of generation the vital nature of hereditary diseases; or in other words, to show that the morbid impression is established upon the vital properties of the ovum, and of course upon those of the new being; and that the hereditary vitiation does not consist in any transmitted impurity to the blood or other fluids of the offspring, as is now supposed by the humoralists, and the fallacy of which I have endeavoured to establish in my Essay on the 'Humoral Pathology.'" (pp. 10, 11.)

We have thus quoted a whole consecutive page of Dr. Paine's lucubrations, in order to prevent any possibility of that misconstruction from imperfect quotation, which we continually encounter in Dr. Paine's criticisms upon his opponents. We must now add, however, that having carefully examined the whole of the Essay, we have failed to discover in it the originality which its author claims; and that we conceive him to have been guided in the line of argument which he adopts, by the delusive conception, that those physiologists who admit the development of the germ to be the consequence of the vital power of the parent (and who does not?) are egregiously in error when they attribute *any* of the processes of the adult being to any other principles whatever. In other words, that, if we admit that organised tissues have vital properties at all, these vital properties must do *all* the work of the organism;—those physiologists being guilty of the most egregious inconsistency, who admit the existence of vital properties as distinct from physical and chemical, and yet imagine that the latter participate in any way in the production of the phenomenon of life. We could almost wish in our hearts that Dr. Paine were suffering under an inflamed leg, in order that he might, by himself experiencing the relief afforded by keeping the limb in the horizontal posture, be convinced that the circulation of the blood is influenced by the force of gravity. That we do not misrepresent his opinions, is evident from the following sentence. "We have thus before us a peculiar order of powers by which the organic being is developed, fashioned, and for ever exclusively governed." Among the adversaries, convicted by his searching comparisons of the most inconsistent and unphilosophical jumble of vitality and chemistry, are Müller, Liebig, Roget, Prout, and Carpenter; the last of whom he styles, "the acknowledged head of the strictly chemical school of physiology,"—a distinction which Dr. Carpenter has, we believe, never courted, and cannot desire, since the existence of peculiar vital properties, and the limited operation of physical and chemical principles, is as distinctly stated in his writings, as in the present article. "But there are none so blind," &c.

The only novel idea which we have encountered in this Essay, is Dr. Paine's comparison of the *germ* to the *gastric juice!* (p. 22.) The following are the assumed points of similarity. "1. The germ is an organic fluid, and so is the gastric juice. 2. The germ possesses a 'creative force,' by which new matter from without is assimilated. Here the analogy is very remarkable, since the gastric juice in bestowing the first vitalizing act upon dead matter must also be peculiarly creative." The first reminds us of Fluellen's celebrated parallel, "There is a river in Macedon," &c. We may dispose of it, however, by assuring Dr. Paine that he is mistaken in supposing the germ to be an organic fluid; since it has been proved, by the aid of the microscope (which vies with the test-tube in its unfortunate tendency to disturb Dr. Paine's peace of mind by the revelation of new facts,) to be the nucleus of a cell; and it has consequently no analogy whatever to the gastric juice. With respect to the second of these comparisons, we simply deny that the gastric juice has any creative power analogous to that of the germ, because none has ever been proved to be manifested by it.

As a kind of Appendix to this Essay is an account of the conclusions derived by Dr. Paine from the presence of nitrogen in organic compounds. These conclusions are chiefly based upon misconceptions of sundry statements contained in Liebig's first work on Organic Chemistry, respecting the tendency of azotized compounds to undergo spontaneous decomposition. These misconceptions seem chiefly the result of ignorance; we can hardly suppose the perversion wilful. Thus Dr. Paine asks, (p. 35,) "by what special art, when the chemist *creates* organic compounds, does he compel nitrogen to undergo a change of its natural characteristic, 'by which' (according to Liebig,) 'it evinces no particular attraction to any one of the simple bodies,' but an absolute repulsion?" The last clause is added by Dr. Paine; and it is as contrary to well-known facts as it is to Liebig's real meaning. For is it to be supposed that nitrogen has a repulsion for oxygen, when every electric discharge passed through a mixture of the two, causes them to unite into nitric acid? What was meant by Liebig was simply this,—that nitrogen has not an attraction for any one element in particular, so that its compounds are easily disturbed. Thus nitric acid is readily deprived of part of its oxygen by anything that has a moderately strong affinity for the latter; and chlorine passed through a solution of ammonia, will detach the nitrogen from the hydrogen, and form with it a new compound, which is again decomposed with violent explosion, whenever it is even brought into contact with oily matter. Hence the unstable nature of azotized compounds is familiar to the inorganic chemist; and there is no more need to suppose the necessity of any peculiar force requisite to bring azote into union with other elements for the sake of producing an organic compound, than there is to imagine that the inorganic chemist requires it. Moreover, the whole of Dr. Paine's argument, founded upon such erroneous assumptions, is refuted by the fact that the tissues of plants are not composed of azotized compounds.

The last Essay, on the Modus Operandi of Remedies, we only here notice as an exemplification of the probability of the advancement of medical science by Dr. Paine's lucubrations. He affirms that "the partial absorption of certain remedies is only a contingent result, and has little or no agency in the physiological phenomena. Their reputed absorption is greatly over-rated, often only imaginary, and sometimes misrepresented." "Whatever is true of remedial agents, in this respect, is equally so of morbific." Thus, to carry out Dr. Paine's principles, the transformation of gouty concretions of uric acid, under the influence of benzoic acid introduced into the stomach, into soluble hippuric acid, which is taken up and carried out of the system, is not an influence of chemical action resulting from the absorption of the benzoic acid and its conveyance to the seat of the deposit, but is merely an example of the operation of forces which (although the operation can be precisely imitated by the chemist, and was indeed suggested in his laboratory) are "transcendentally vital." Of a verity these dogmas altogether transcend our poor comprehension; and it will not be on slight grounds that we shall again call the attention of our readers to such an involved concatenation of absurdities.

---

### Art. XIII.

*Medico-Chirurgical Transactions.* Vol. XXV.—*London*, 1842. 8vo, pp. 319. With 7 Plates.

